# Association of Bone-Specific Physical Activity with Bone Mineral Density and Arterial Stiffness in Premenopausal Women: A Cross-Sectional Study

**DOI:** 10.3390/healthcare14193287

**Published:** 2026-10-02

**Authors:** Sung-Woo Kim, Myong-Won Seo, Hyun Chul Jung

**Affiliations:** 1 Department of Sports Medicine and Science, Graduate School, Konkuk University, 120 Neungdong-ro, Gwangjin-gu, Seoul 05029, Republic of Korea; kswrha@konkuk.ac.kr; 2 Physical Activity and Performance Institute, Konkuk University, 120 Neungdong-ro, Gwangjin-gu, Seoul 05029, Republic of Korea; 3 Department of Sports and Leisure Studies, College of Physical Education, Keimyung University, 1095 Dalgubeol-Daero, Dalseo-gu, Daegu 42601, Republic of Korea; myongwonseo@kmu.ac.kr; 4 Department of Sports Coaching, College of Physical Education, Kyung Hee University, 1732 Deogyeong-Daero, Giheung-gu, Yongin 17104, Gyeonggi-do, Republic of Korea

**Keywords:** bone-specific physical activity, bone health, pulse wave velocity, vascular health, premenopausal women

## Abstract

**Background:** This cross-sectional study investigated the associations of bone-specific physical activity (PA), assessed using the Bone-Specific Physical Activity Questionnaire (BPAQ), with bone mineral density (BMD) and arterial stiffness in premenopausal women. **Methods:** A total of 270 premenopausal women participated. Continuous total BPAQ (tBPAQ) was analyzed as the primary exposure in the revised analyses, while tertile-based analyses were retained as secondary analyses. BMD was measured by dual-energy X-ray absorptiometry, and arterial stiffness was assessed using brachial-ankle pulse wave velocity (baPWV). BMD models were adjusted for age, fat-free mass (FFM), and BMI, and the mean bilateral baPWV model was adjusted for age, mean arterial pressure, and BMI. **Results:** Higher tBPAQ was positively associated with whole-body, femoral neck, total femur, and lumbar spine BMD after false-discovery-rate correction (all q < 0.001). The forearm association was weaker (B = 0.0040 g/cm^2^ per 10 tBPAQ points; q = 0.019) and was not retained in the log1p-transformed sensitivity analysis (*p* = 0.162) or the adjusted tertile comparison (*p* = 0.062). There was no evidence of an adjusted linear association between tBPAQ and mean bilateral baPWV (B = 5.1 cm/s per 10 points; 95% CI, −4.6 to 14.7; *p* = 0.301; q = 0.301). Exploratory quadratic models suggested possible nonlinearity for femoral neck BMD and mean bilateral baPWV, but these findings were hypothesis-generating. **Conclusions:** Higher cumulative bone-specific PA was associated with higher BMD, particularly at weight-bearing skeletal sites, whereas the forearm finding was less consistent across analyses. No adjusted linear association with arterial stiffness was identified. Longitudinal and interventional studies are needed to clarify temporality and possible nonlinear relationships.

## 1. Introduction

Cardiovascular disease (CVD) remains the leading cause of mortality worldwide, and its prevalence continues to increase annually [1]. Accumulating evidence suggests that skeletal and vascular health are biologically interconnected. Low bone mineral density (BMD) has been associated with vascular calcification, arterial stiffness, atherosclerosis, and increased cardiovascular risk, indicating that bone loss and vascular dysfunction may share common pathophysiological pathways [2,3,4,5,6,7,8,9].

Regular physical activity (PA) is recognized as a key non-pharmacological strategy for the prevention of both osteoporosis and CVD. Long-term engagement in PA improves bone mass, vascular function, and cardiometabolic health while reducing the risk of chronic diseases, including obesity, hypertension, and diabetes [10]. Despite these benefits, physical inactivity remains highly prevalent worldwide, with approximately one-third of adults failing to meet recommended activity levels, and the prevalence of inactivity increases with age [11,12]. These findings highlight the importance of promoting appropriate PA across the lifespan to preserve both skeletal and cardiovascular health.

High-impact, weight-bearing activities such as jumping and running generate mechanical loading that is particularly relevant to bone formation and maintenance [13,14]. Mechanical loading is central to skeletal adaptation, while repeated physical activity also produces hemodynamic stimuli that may influence vascular function [15]. These parallel mechanical and circulatory responses provide a rationale for examining bone-specific physical activity in relation to both skeletal and vascular outcomes. However, most previous studies have focused on general or aerobic PA and have used questionnaires that do not specifically characterize osteogenic loading [16,17,18]. 

The Bone-Specific Physical Activity Questionnaire (BPAQ) was developed to quantify lifetime exposure to osteogenic loading based on ground reaction forces and mechanical loading [19]. BPAQ is a more sensitive predictor of BMD than traditional PA measures [20,21,22], and previous work has suggested associations with cardiovascular risk profiles [20]. However, evidence regarding BPAQ-derived bone-specific PA and arterial stiffness remains limited, particularly in premenopausal women. Therefore, this cross-sectional study examined the associations of BPAQ-derived bone-specific PA with BMD and arterial stiffness in young and middle-aged premenopausal women. We hypothesized that higher BPAQ scores would be associated with higher BMD and lower arterial stiffness.

## 2. Materials and Methods

### 2.1. Participants

**Figure 1:**
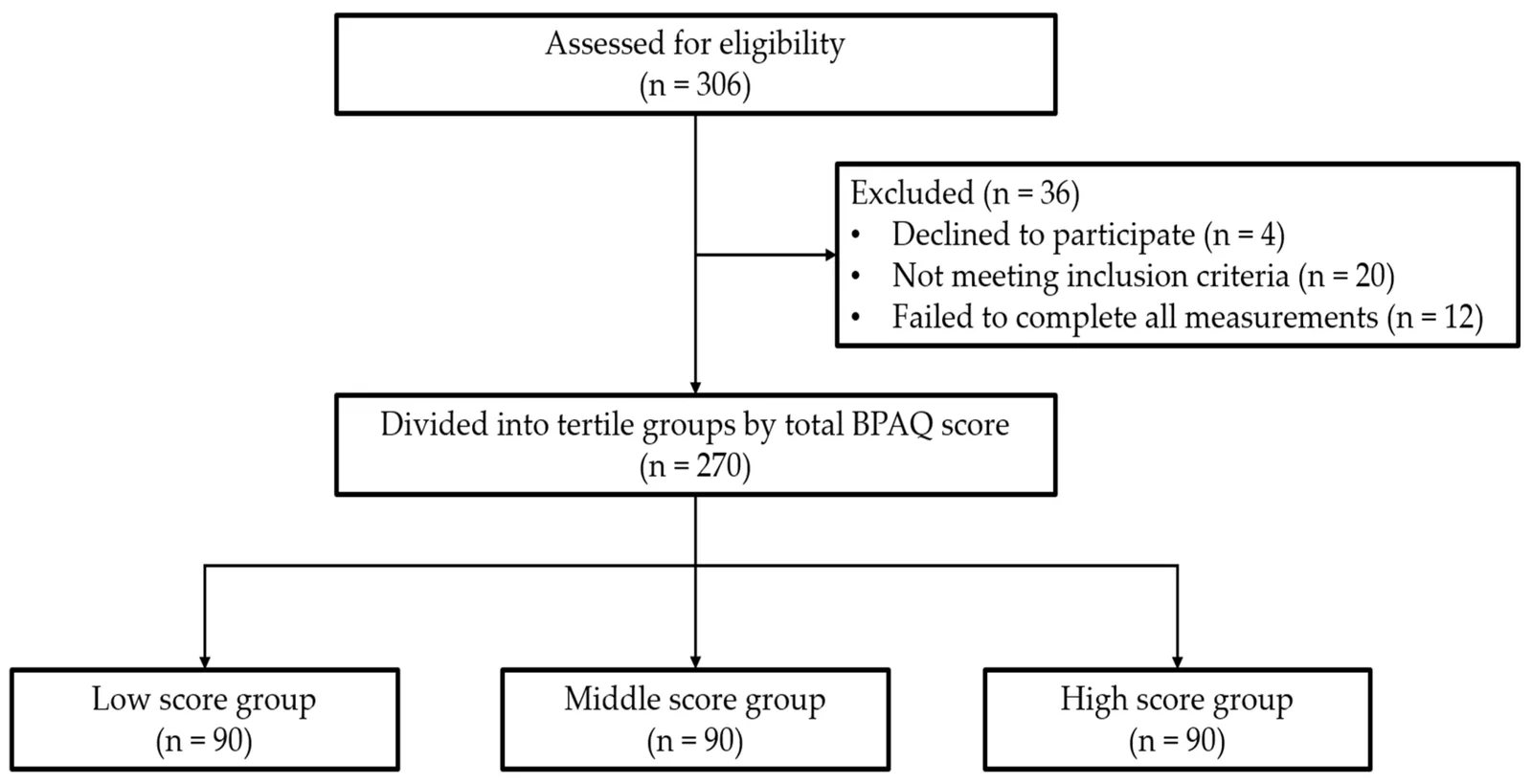
Study flowchart.

Initially, 306 young and middle-aged premenopausal women volunteered for the study. The inclusion criterion was premenopausal status. The exclusion criteria were the use of pharmaceutical agents that directly affect bone metabolism and the use of medications for hypertension or hyperlipidemia. Participants were recruited between January and May 2018 through recruitment posters. Premenopausal status was confirmed based on the absence of a previous medical diagnosis of menopause at a hospital. Pregnant women were excluded from participation. Thirty-six participants were excluded for declining to participate, not meeting the inclusion criteria, or failing to complete all measurements; thus, 270 participants completed the study (Figure 1). The final analytical sample ranged in age from 18.3 to 55.4 years. For secondary tertile analyses, participants were ranked by tBPAQ and categorized into low, middle, and high groups (*n* = 90 per group). Participants received detailed explanations of the study objectives and procedures, including their benefits and potential risks, and informed consent was obtained from each participant. The basic characteristics of the participants are summarized in Table 1.

### 2.2. Study Procedures

All measurements were conducted at the Growth and Aging Laboratory of Kyunghee University and followed a standardized testing protocol. Participants attended the laboratory on a single visit and were instructed to refrain from strenuous PA, alcohol consumption, caffeine intake, and smoking for at least 24 hours before testing. Upon arrival, participants completed an informed consent form and the BPAQ. Anthropometric measurements were obtained first, followed by assessments of body composition and BMD and arterial stiffness. Participants wore light clothing and removed all metallic objects before testing. All measurements were performed by trained research personnel.

### 2.3. Physique Measurement

Body height was measured using a stadiometer to the nearest 0.1 cm (T.K.K. 11253, Takei Scientific Ins Co., Tokyo, Japan). Body weight was measured using a digital scale (150A, CAS, Seoul, Republic of Korea) to the nearest 0.1 kg. Body mass index (BMI) was calculated by dividing body weight (kg) by the square of body height (m^2^).

### 2.4. Body Composition and Bone Mineral Density

Body composition and BMD in the whole body, femoral neck, total femur, lumbar spine, and forearm were assessed using dual-energy X-ray absorptiometry (DXA) with a Hologic QDR 4500W bone densitometer (Hologic Inc., Marlborough, MA, USA). A single trained technician performed all scans to minimize inter-operator variability. Whole-body DXA analysis provided fat mass (FM), percent body fat (%BF), and fat-free mass (FFM). The FFM variable used in the present analyses did not include bone mineral content, which was quantified separately by DXA. Lumbar spine BMD was measured at L1–L4 and expressed as the mean BMD across these vertebrae. Femoral BMD was assessed at the left proximal femur, including the femoral neck and total femur regions, with the leg internally rotated by approximately 15–20° to ensure appropriate femoral neck alignment. Forearm BMD was measured at the one-third (33%) distal radius of the non-dominant forearm. Regions of interest were defined using the manufacturer’s analysis software and visually inspected by the trained technician, with manual adjustments as needed. Instrument quality control was performed in accordance with the manufacturer’s recommendations using the Hologic spine phantom, and participant scans were conducted only after the quality-control procedure was successfully completed. The same densitometer and standardized acquisition and analysis procedures were used throughout the study period.

### 2.5. Arterial Stiffness

Blood pressure and brachial-ankle pulse wave velocity (baPWV) were assessed using an automated waveform analyzer (VP-1000, Colin Co., Komaki, Japan). Systolic blood pressure (SBP), diastolic blood pressure (DBP), and mean arterial pressure (MAP) obtained during the assessment were used for subsequent analyses. All measurements were performed in a quiet, temperature-controlled room to minimize external influences on vascular tone.

Participants rested in a supine position for at least 10 min before measurement. Electrocardiographic electrodes were placed on both wrists, a phonocardiographic microphone was positioned at the left sternal border, and pressure-sensor-equipped cuffs were applied to both upper arms and ankles. The device simultaneously recorded arterial pressure waveforms and automatically calculated right and left baPWV values from the estimated arterial path length and pulse transit time. Two consecutive baPWV measurements were obtained for each participant. For each side, the two measurements were averaged to obtain the final right- and left-baPWV values. For the revised analyses, mean bilateral baPWV was subsequently calculated as the average of the final right and left values and was designated as the principal vascular outcome.

### 2.6. High-Impact Physical Activity

High-impact PA was assessed using the BPAQ [19]. Current BPAQ (cBPAQ) represents bone-relevant physical activity during the 12 months preceding assessment, whereas past BPAQ (pBPAQ) represents earlier lifetime exposure; total BPAQ (tBPAQ) integrates current and past exposure according to the established BPAQ scoring procedure. To improve measurement reliability, a one-to-one interview was conducted with each participant, and responses were processed using the BPAQ scoring program. For secondary tertile analyses, participants were rank-ordered by tBPAQ and divided into three equal-sized groups (*n* = 90 each). The observed maximum tBPAQ value in the low group was 2.6620, the observed middle-group range was 2.6625–12.2570, and the observed minimum value in the high group was 12.6585. These values represent observed order statistics rather than prespecified continuous classification cutoffs.

### 2.7. Statistical Analysis

Statistical analyses were conducted using IBM SPSS Statistics for Windows, version 28.0 (IBM Corp., Armonk, NY, USA). Continuous variables are presented as mean ± standard deviation (SD) and 95% confidence intervals (CIs) where applicable. Because BPAQ distributions were right-skewed, cBPAQ, pBPAQ, and tBPAQ were additionally summarized as median [interquartile range (IQR)]. Distributional characteristics were evaluated using skewness and the Kolmogorov–Smirnov test with the Lilliefors correction. During revision, continuous tBPAQ was designated as the primary exposure to reduce reliance on categorization; cBPAQ, pBPAQ, and tBPAQ tertiles were retained as secondary analyses. Participant characteristics were compared using one-way analysis of variance (ANOVA). Body composition outcomes were assessed using age-adjusted analysis of covariance (ANCOVA). For the BMD domain, multivariate analysis of covariance (MANCOVA) was performed for five BMD outcomes with age, FFM, and BMI as covariates, followed by outcome-specific ANCOVA. Bilateral baPWV was evaluated using MANCOVA with age, right-brachial MAP, and BMI as covariates; during revision, mean bilateral baPWV was designated as the principal vascular outcome. General linear models used Type III sums of squares, and pairwise estimated marginal mean comparisons were Bonferroni-adjusted. Primary multivariable linear regression models were performed using the Enter method to examine the association between continuous tBPAQ and each outcome, with tBPAQ scaled so that the regression coefficient represents the change in the outcome per 10-point increase in tBPAQ. BMD models included age, FFM, and BMI; the vascular model included age, right-brachial MAP, and BMI. Unstandardized coefficients (B), 95% CIs, standardized beta coefficients (β), and adjusted R^2^ were reported. The six-outcome multiplicity family (five BMD outcomes and mean bilateral baPWV) was defined for the revised primary regression analysis, and Benjamini–Hochberg false-discovery-rate (FDR) correction was applied across these six raw *p*-values. Related secondary outcome families were corrected separately where specified. The secondary exposure regressions in Supplementary Table S4 were exploratory and were not included in the primary six-outcome FDR family; nominal *p*-values are reported for those analyses. Spearman correlations and age-adjusted partial correlations supplemented Pearson correlations. Sensitivity analyses included log1p(tBPAQ), defined as ln(1 + tBPAQ), and mean-centered quadratic tBPAQ terms. Regression diagnostics included tolerance and variance inflation factors (VIFs), standardized/studentized residuals, residual-versus-predicted plots, leverage, and Cook’s distance. Case-deletion sensitivity analyses excluded observations with |studentized residual| > 3. For the two nominally significant quadratic terms, an additional sensitivity analysis excluded the three highest observed tBPAQ values. Partial eta-squared (ηp^2^) was reported for group comparisons. Statistical significance was set at *p* < 0.05, with FDR-adjusted q-values used for multiplicity-controlled primary inference. As a descriptive sample-size sensitivity analysis for a simple two-sided Pearson correlation, a sample size of 270 provides approximately 80% power at α = 0.05 to detect a correlation of approximately |r| = 0.169. This calculation was not used to infer statistical power for the multivariable, multiplicity-controlled, or nonlinear analyses.

## 3. Results

### 3.1. Body Composition

Participant characteristics are summarized in Table 1. Age differed among tBPAQ tertiles (F = 3.178, *p* = 0.043, ηp^2^ = 0.023), whereas body height, body weight, and BMI did not differ significantly. BPAQ distributions were strongly right-skewed, particularly cBPAQ, and are therefore reported using both mean ± SD and median [IQR]. In age-adjusted secondary analyses of body composition (Table 2), FFM differed among groups (F = 6.877, *p* = 0.001, ηp^2^ = 0.049; FDR q = 0.004), with higher adjusted FFM in the high group than in both the low and middle groups in Bonferroni-adjusted pairwise comparisons. Percent body fat showed a nominal group effect (*p* = 0.036) but did not remain significant after FDR correction (q = 0.053); fat mass did not differ significantly (*p* = 0.421).

### 3.2. Bone Mineral Density

The adjusted multivariate group effect for the five BMD outcomes was significant (Pillai’s trace = 0.1189, F(10, 522) = 3.300, *p* < 0.001). Follow-up ANCOVA adjusted for age, FFM, and BMI showed significant group effects for whole-body BMD (F = 9.079, *p* < 0.001, q < 0.001, ηp^2^ = 0.064), femoral neck BMD (F = 9.990, *p* < 0.001, q < 0.001, ηp^2^ = 0.070), total femur BMD (F = 14.122, *p* < 0.001, q < 0.001, ηp^2^ = 0.097), and lumbar spine BMD (F = 9.010, *p* < 0.001, q < 0.001, ηp^2^ = 0.064). The adjusted forearm BMD group effect was not significant (F = 2.818, *p* = 0.062, q = 0.062, ηp^2^ = 0.021). Bonferroni-adjusted estimated marginal mean comparisons indicated that the high group had higher whole-body, femoral neck, total femur, and lumbar spine BMD than the low group; the high group also exceeded the middle group for these outcomes (Table 3).

### 3.3. Arterial Stiffness

In unadjusted secondary analyses, mean bilateral baPWV decreased across the low-, middle-, and high-tBPAQ groups (1155.5 ± 142.1, 1139.3 ± 182.3, and 1099.0 ± 138.1 cm/s, respectively; F = 3.152, *p* = 0.044). However, the adjusted multivariate group effect for bilateral baPWV was not significant after controlling for age, right-brachial MAP, and BMI (Pillai’s trace = 0.0070, F(4, 528) = 0.467, *p* = 0.760). Follow-up ANCOVA for mean bilateral baPWV was also not significant (F = 0.281, *p* = 0.755, ηp^2^ = 0.002), indicating that the unadjusted vascular pattern was attenuated after covariate adjustment (Table 4).

### 3.4. Adjusted Associations of BPAQ with BMD and Arterial Stiffness

In the revised primary continuous-exposure analyses, higher tBPAQ remained positively associated with BMD after adjustment for age, FFM, and BMI (Table 5). Per 10-point increase in tBPAQ, whole-body BMD was higher by 0.0178 g/cm^2^ (95% CI, 0.0113–0.0244; β = 0.301; *p* < 0.001; q < 0.001), femoral neck BMD by 0.0331 g/cm^2^ (95% CI, 0.0230–0.0431; β = 0.348; *p* < 0.001; q < 0.001), total femur BMD by 0.0314 g/cm^2^ (95% CI, 0.0228–0.0400; β = 0.382; *p* < 0.001; q < 0.001), lumbar spine BMD by 0.0236 g/cm^2^ (95% CI, 0.0137–0.0335; β = 0.271; *p* < 0.001; q < 0.001), and forearm BMD by 0.0040 g/cm^2^ (95% CI, 0.0008–0.0072; β = 0.145; *p* = 0.015; q = 0.019). The forearm finding was less consistent across alternative specifications: the adjusted tertile comparison was not significant (*p* = 0.062), and the log1p-transformed sensitivity model was also not significant (*p* = 0.162). There was no evidence of an adjusted linear association between tBPAQ and mean bilateral baPWV (B = 5.1 cm/s per 10 tBPAQ points; 95% CI, −4.6 to 14.7; β = 0.043; *p* = 0.301; q = 0.301).

Regression diagnostics did not indicate material multicollinearity: the maximum VIF was 1.861 across the BMD models and 1.351 in the vascular model. One or two observations per primary model had |studentized residual| > 3, but the maximum Cook’s distance across all primary models was 0.140, and no Cook’s distance approached 1. Residual-versus-predicted plots did not show severe systematic patterns. Excluding observations with |studentized residual| > 3 did not alter the primary inference: the tBPAQ coefficient remained significant for all five BMD outcomes (including forearm BMD, B = 0.0036, *p* = 0.024) and remained nonsignificant for mean bilateral baPWV (B = 6.6 cm/s, *p* = 0.158).

Secondary analyses showed positive adjusted associations of pBPAQ with the BMD outcomes, whereas cBPAQ showed weaker and less consistent adjusted associations. Age-adjusted partial correlations markedly attenuated the inverse correlations between BPAQ-baPWV and PWV. Spearman and log1p-transformed analyses generally supported the principal skeletal findings, with the forearm exception noted above. In exploratory quadratic models using tBPAQ/10 centered at the sample mean, femoral neck BMD showed a positive centered linear term (B = 0.0202; 95% CI, 0.0051–0.0354; *p* = 0.009) and a positive quadratic term (B = 0.00526; 95% CI, 0.00060–0.00993; *p* = 0.027; adjusted R^2^ = 0.300). For mean bilateral baPWV, the centered linear term was not significant (B = −7.79; 95% CI, −22.03 to 6.46; *p* = 0.283), whereas the quadratic term was positive (B = 5.35; 95% CI, 0.94–9.75; *p* = 0.017; adjusted R^2^ = 0.592). Excluding the three highest observed tBPAQ values did not eliminate these quadratic terms (femoral neck BMD: quadratic B = 0.00926, *p* = 0.004; mean bilateral baPWV: quadratic B = 6.82, *p* = 0.026). Because these nonlinear analyses were exploratory and were not part of the primary multiplicity-controlled analysis, they are interpreted as hypothesis-generating.

## 4. Discussion

The present study examined the associations of BPAQ-derived bone-specific PA with body composition, BMD, and arterial stiffness in premenopausal women. The principal finding was that greater cumulative bone-specific PA, represented by tBPAQ, was positively associated with BMD at the whole body, femoral neck, total femur, lumbar spine, and forearm after adjustment for age, FFM, and BMI and after correction for multiple testing. The magnitudes of the adjusted associations were greatest for the total femur and femoral neck and more modest for the forearm; moreover, the forearm association was not retained in the tertile- or log-transformed sensitivity analyses. In contrast, although unadjusted analyses suggested lower baPWV among women with higher BPAQ scores, there was no evidence of a linear association after adjusting for age, MAP, and BMI. Thus, the present findings provide stronger and more consistent support for a skeletal association than for a linear vascular association.

Body composition, including FFM and FM, can be improved through lifestyle changes, such as increased PA and better control of nutrient intake, in young and middle-aged women [23,24]. Generally, body composition changes with age and a prolonged sedentary lifestyle, toward increased adipose tissue and decreased FFM [25,26]. Although it is well known that regular PA increases FFM and decreases %BF, these effects are small in women [27]. Unlike previous studies, the current study found no significant differences in FM and %BF among the three groups. However, our study showed that FFM in the high-BPAQ score group was significantly higher than that of the low-BPAQ score group. These results are consistent with previous studies. Saravi & Sayegh reported that FFM in the habitual PA group was significantly higher than that in the sedentary group in premenopausal women when measured by PA levels using the International Physical Activity Questionnaire [28]. Moreover, Guo et al. reported that higher PA is associated with lower adiposity in both premenopausal and postmenopausal women in their large cross-sectional study of 132,793 British women [29]. In the present study, age-adjusted FFM was significantly higher in the high-tBPAQ group than in the low and middle groups, whereas fat mass did not differ significantly and the nominal difference in percent body fat did not remain significant after correction for multiple testing. These findings suggest that the body composition component most consistently associated with high bone-specific activity in this cohort was lean mass rather than fat mass.

The higher FFM observed among women with greater tBPAQ may also be physiologically relevant to skeletal adaptation. Muscle-generated forces are an important source of mechanical loading on bone [13], and weight-bearing and impact activities provide osteogenic stimuli that help maintain bone mass [14]. Greater muscle mass may therefore increase the magnitude and frequency of mechanical forces transferred to the skeleton. Importantly, however, the present associations between tBPAQ and BMD remained significant after statistical adjustment for FFM and BMI. This suggests that differences in measured body composition alone did not account for the association between cumulative bone-specific PA and BMD. At the same time, FFM may lie partly along the biological pathway linking habitual loading to skeletal adaptation; consequently, the adjusted associations should not be interpreted as demonstrating an effect entirely independent of muscle-mediated mechanical loading.

The present skeletal findings are broadly consistent with previous studies examining habitual and bone-specific PA. Physically active premenopausal women have been reported to have greater BMD than women with lower activity levels [28], and longitudinal evidence indicates that PA performed during adulthood is positively associated with BMD many years later [30]. Studies using the BPAQ have similarly demonstrated associations between bone-specific PA and bone mineral density, strength, and geometry in different adult populations [20,21,22,31,32]. Weeks et al. reported higher lumbar BMD among individuals in the upper BPAQ tertile compared with those in the lower tertile [20], while Bolam et al. demonstrated associations between BPAQ-derived activity and bone density in middle-aged and older adults [21]. Previous studies in young and middle-aged women have also linked higher bone-specific PA scores with favorable bone geometry, strength, body composition, and BMD [22,31,32]. The present study extends these observations by showing that the relationship between cumulative BPAQ exposure and BMD persisted after simultaneous adjustment for age, FFM, and BMI, and after controlling the false discovery rate across multiple skeletal outcomes.

An additional finding was that past and cumulative BPAQ exposure showed more consistent skeletal associations than current BPAQ. This pattern is biologically plausible because BMD reflects skeletal adaptation accumulated over a prolonged period rather than exposure during only a short recent interval. Historical and longitudinal studies have demonstrated that PA across earlier life and adulthood contributes to later bone characteristics [16,17,18,30]. Nevertheless, the weaker associations observed for cBPAQ should not be interpreted as evidence that recent exercise is ineffective for bone. Reviews of exercise and bone health support the osteogenic importance of appropriately loaded PA [33,34], and intervention evidence in middle-aged premenopausal women indicates that high-impact weight-bearing exercise can modify bone-related outcomes [35]. Rather, in the context of a cross-sectional study, pBPAQ and tBPAQ may better capture the cumulative mechanical loading history reflected in current BMD than an activity score limited to the preceding 12 months.

The site-specific results also deserve consideration. The strongest standardized associations with tBPAQ were observed at the total femur and femoral neck, followed by whole-body and lumbar spine BMD, whereas the association with forearm BMD was considerably smaller. This pattern is consistent with the site-specific nature of mechanical loading, as many high-impact weight-bearing activities captured by the BPAQ predominantly load the lower extremities and axial skeleton [13,14,19]. Although forearm BMD remained significantly associated with tBPAQ in the primary adjusted model after FDR correction, the association was not significant in the log-transformed sensitivity analysis. Accordingly, the forearm result appears less robust than the findings from weight-bearing skeletal sites and should be interpreted with caution. More generally, the marked right-skew of BPAQ scores emphasizes the importance of considering both conventional and sensitivity analyses when evaluating dose–response relationships.

The vascular findings differed substantially from the skeletal findings. PA is widely recognized as an important modifier of vascular health [36], and observational studies have reported inverse relationships between habitual PA and arterial stiffness. Aoyagi et al. found that greater habitual activity was associated with less regional arterial stiffening in older adults [37], while Gando et al. reported lower arterial stiffness among older adults who accumulated more light-intensity PA [38]. BPAQ-derived PA has also previously been associated with cardiovascular risk profiles [20]. Consistent with these observations, our unadjusted analyses initially showed lower baPWV among participants with higher tBPAQ. However, the association was attenuated after adjustment for age, MAP, and BMI: neither the bilateral baPWV MANCOVA, covariate-adjusted group comparison, age-adjusted partial correlations nor the primary multivariable regression supported an independent linear association between tBPAQ and mean bilateral baPWV.

Several factors may explain the difference between the unadjusted and adjusted vascular findings. First, age differed significantly among the BPAQ groups in the present dataset, and blood pressure was directly incorporated into the adjusted vascular models. The loss of association after controlling for these variables suggests that at least part of the unadjusted BPAQ–baPWV relationship reflected differences in participant characteristics rather than an independent relationship with bone-specific PA. Second, the BPAQ was developed primarily to characterize osteogenic mechanical loading rather than the cardiovascular dose of exercise [19]. It does not directly quantify aerobic exercise volume, sustained metabolic intensity, cardiorespiratory fitness, or cumulative vascular shear-stress exposure. Therefore, a high BPAQ score does not necessarily represent the type or amount of activity most strongly associated with arterial adaptation.

This distinction is relevant because the vascular effects of exercise may depend on exercise modality. Reviews indicate that aerobic, resistance, and combined training may have different effects on arterial stiffness [39], and combined aerobic and resistance training does not necessarily produce the same arterial response as aerobic training alone [40]. Thus, the absence of an adjusted linear association between tBPAQ and baPWV in the present study does not conflict with the broader literature demonstrating cardiovascular benefits of regular PA [36,41,42]. Rather, it indicates that cumulative osteogenic loading, as quantified by BPAQ, should not be assumed to have a simple linear relationship with arterial stiffness in relatively healthy premenopausal women. The exploratory quadratic model suggested a shallow curvilinear pattern in mean bilateral baPWV, whereas the femoral neck model indicated increasing BMD at higher tBPAQ values. These patterns persisted after excluding the three highest tBPAQ observations, but exposure values were sparse at the upper end of the distribution and confidence intervals widened accordingly. Because the quadratic analyses were adopted during revision, were not multiplicity-controlled as primary tests, and were not based on an a priori nonlinear hypothesis, they should be regarded as hypothesis-generating rather than evidence of established nonlinear dose–response relationships.

The distinction between skeletal and vascular findings is particularly important when considering the broader health implications of PA. Physical inactivity contributes substantially to the global burden of non-communicable disease [41], and regular PA remains a fundamental component of health promotion [42]. Nevertheless, the present cross-sectional results do not provide evidence that high BPAQ exposure itself reduces arterial stiffness or cardiovascular risk. Instead, they support a more specific interpretation: sustained participation in activities that impose osteogenic loading is associated with more favorable skeletal characteristics in premenopausal women, whereas the apparent vascular association is largely attenuated after accounting for major hemodynamic and demographic covariates. Accordingly, the current results should not be used to infer a common “dual protective effect” of high-impact activity on bone and the vasculature.

## 5. Limitation of the Study

This study has several limitations. First, the cross-sectional design precludes determination of temporality or causality, and reverse causation remains possible because women with more favorable musculoskeletal health may be more willing or able to participate in high-impact activity. Second, BPAQ is self-reported, and pBPAQ requires retrospective recall over many years; recall accuracy may therefore vary by age. Third, reproductive and hormonal characteristics, including menstrual-cycle phase, hormonal contraceptive use, parity, pregnancy/lactation history, and circulating sex-hormone concentrations, were not systematically available. Habitual calcium and vitamin D intake or supplementation, smoking, and habitual alcohol consumption were also not systematically quantified. These unmeasured factors may produce residual confounding of both skeletal and vascular associations.

## 6. Conclusions

This cross-sectional study indicates that higher cumulative bone-specific PA is associated with higher BMD in premenopausal women after adjustment for age, body composition, and multiple testing. Associations at the whole body, femoral neck, total femur, and lumbar spine were more consistent across analyses, whereas the forearm finding was weaker and less robust to alternative specifications. There was no evidence of an adjusted linear association between tBPAQ and mean bilateral baPWV. Exploratory quadratic models suggested possible nonlinear patterns for femoral neck BMD and mean bilateral baPWV, but these findings require independent confirmation and do not establish causality. Longitudinal and interventional studies incorporating detailed reproductive, hormonal, dietary, and cardiovascular covariates are needed to determine temporality and clinical relevance.

## Figures and Tables

**Table 1:** Physical characteristics and physical activity level.

Variables	All-Subject (*n* = 270)	Low-Score Group (*n* = 90)	Middle-Score Group (*n* = 90)	High-Score Group (*n* = 90)	F-Value	Prob > F
Age (years)	35.97 ± 8.40	37.22 ± 6.96	36.48 ± 9.08	34.21 ± 8.79	3.178	0.043
Body height (cm)	161.62 ± 5.10	161.21 ± 4.93	161.37 ± 4.88	162.29 ± 5.47	1.169	0.312
Body weight (kg)	58.65 ± 9.11	57.55 ± 8.68	58.80 ± 9.76	59.59 ± 8.85	1.143	0.320
BMI (kg/m^2^)	22.43 ± 3.19	22.12 ± 3.03	22.58 ± 3.60	22.58 ± 2.90	0.617	0.540
Current BPAQ (score)	3.31 ± 8.43 0.56 [0.00–2.54]	0.37 ± 0.55 0.15 [0.00–0.56]	1.88 ± 2.95 0.62 [0.07–2.35]	7.69 ± 13.25 2.19 [0.07–9.59]	—	—
Past BPAQ (score)	19.53 ± 23.28 10.24 [2.47–31.09]	2.13 ± 1.23 1.88 [1.29–2.84]	11.37 ± 6.35 10.85 [6.55–16.31]	45.07 ± 23.73 40.97 [31.45–51.72]	—	—
Total BPAQ (score)	11.41 ± 13.20 6.17 [1.72–18.17]	1.23 ± 0.67 1.09 [0.70–1.69]	6.63 ± 2.76 6.17 [4.17–8.66]	26.38 ± 12.80 22.19 [18.24–30.14]	—	—

Note: Values are expressed as mean ± SD. BPAQ variables are additionally reported as median [IQR] because of right-skewed distributions. BMI, body mass index; BPAQ, Bone-Specific Physical Activity Questionnaire. Participants were rank-ordered by tBPAQ and divided into three equal-sized groups (*n* = 90 each). The observed maximum tBPAQ in the low group was 2.6620, the observed middle-group range was 2.6625–12.2570, and the observed minimum in the high group was 12.6585. These values are observed order statistics rather than prespecified classification cutoffs. Inferential testing of BPAQ scores across tBPAQ-defined groups was omitted to avoid circular inference.

**Table 2:** Age-adjusted comparison of body composition among the three groups.

Variables	Low Adjusted Mean (95% CI)	Middle Adjusted Mean (95% CI)	High Adjusted Mean (95% CI)	F-Value	Prob > F	ηp^2^	FDR q
Fat mass (kg)	17.71 (16.55–18.86)	18.69 (17.55–19.84)	17.81 (16.66–18.97)	0.868	0.421	0.006	0.421
Fat-free mass (kg)	36.61 (35.67–37.55)	36.97 (36.04–37.91)	38.95 (38.01–39.90)	6.877	0.001	0.049	0.004
Percent body fat (%)	30.89 (29.74–32.04)	31.78 (30.63–32.92)	29.64 (28.48–30.79)	3.377	0.036	0.025	0.053

Note: Values are age-adjusted estimated marginal means (95% CI) from general linear models using Type III sums of squares. FDR q-values were calculated using the Benjamini–Hochberg procedure across the three body composition outcomes. Bonferroni-adjusted pairwise comparisons showed higher FFM in the high group than in the low and middle groups. CI, confidence interval; FFM, fat-free mass.

**Table 3:** Covariate-adjusted BMD comparisons among total BPAQ groups.

Variables	Low Adjusted Mean (95% CI)	Middle Adjusted Mean (95% CI)	High Adjusted Mean (95% CI)	F-Value	Prob > F	ηp^2^	FDR q
Whole-body BMD (g/cm^2^)	1.110 (1.095–1.124)	1.109 (1.095–1.124)	1.150 (1.135–1.165)	9.079	<0.001	0.064	<0.001
Femoral neck BMD (g/cm^2^)	0.811 (0.788–0.834)	0.805 (0.782–0.828)	0.874 (0.850–0.897)	9.990	<0.001	0.070	<0.001
Total femur BMD (g/cm^2^)	0.860 (0.840–0.879)	0.862 (0.843–0.882)	0.928 (0.908–0.948)	14.122	<0.001	0.097	<0.001
Lumbar spine BMD (g/cm^2^)	0.984 (0.962–1.006)	0.999 (0.977–1.021)	1.050 (1.028–1.072)	9.010	<0.001	0.064	<0.001
Forearm BMD (g/cm^2^)	0.558 (0.550–0.565)	0.552 (0.545–0.559)	0.564 (0.557–0.571)	2.818	0.062	0.021	0.062

Note: Values are covariate-adjusted estimated marginal means (95% CI) from general linear models using Type III sums of squares. BMD models were adjusted for age, fat-free mass, and BMI. FDR q-values were calculated using the Benjamini–Hochberg procedure across the five follow-up BMD outcomes, and pairwise estimated marginal mean comparisons used Bonferroni adjustment. The overall BMD MANCOVA was significant (Pillai’s trace = 0.1189, F(10, 522) = 3.300, *p* < 0.001). BMD, bone mineral density; CI, confidence interval.

**Table 4:** Blood pressure and covariate-adjusted mean bilateral baPWV comparisons among total BPAQ groups.

Variables	Low-Score Group (95% CI)	Middle-Score Group (95% CI)	High-Score Group (95% CI)	F-Value	Prob > F	ηp^2^
Right-brachial SBP (mmHg)	114.0 ± 10.9	114.1 ± 13.9	112.4 ± 11.1	0.532	0.588	0.004
Right-brachial MAP (mmHg)	86.8 ± 10.2	86.5 ± 11.7	83.8 ± 10.7	2.101	0.124	0.015
Right-brachial DBP (mmHg)	67.8 ± 9.1	67.5 ± 9.7	65.0 ± 9.0	2.548	0.080	0.019
Mean bilateral baPWV (cm/s)	1137.6 (1116.4–1158.8)	1129.7 (1108.7–1150.8)	1126.4 (1105.2–1147.7)	0.281	0.755	0.002

Note: Blood-pressure values are unadjusted mean ± SD. Mean bilateral baPWV values are covariate-adjusted marginal means (95% CI) from ANCOVA adjusted for age, right-brachial MAP, and BMI. The bilateral baPWV MANCOVA was not significant (Pillai’s trace = 0.0070, F(4, 528) = 0.467, *p* = 0.760). Right- and left-side baPWV results are retained in the Supplementary Materials.

**Table 5:** Multivariable associations of total BPAQ with BMD and mean bilateral baPWV.

Variables	B per 10-Point tBPAQ	95% CI	Standardized β	*p*-Value	FDR q	Adjusted R^2^
Whole-body BMD	0.0178	0.0113 to 0.0244	0.301	<0.001	<0.001	0.224
Femoral neck BMD	0.0331	0.0230 to 0.0431	0.348	<0.001	<0.001	0.290
Total femur BMD	0.0314	0.0228 to 0.0400	0.382	<0.001	<0.001	0.304
Lumbar spine BMD	0.0236	0.0137 to 0.0335	0.271	<0.001	<0.001	0.189
Forearm BMD	0.0040	0.0008 to 0.0072	0.145	0.015	0.019	0.134
Mean bilateral baPWV	5.1	−4.6 to 14.7	0.043	0.301	0.301	0.585

Note: Values were obtained from Enter-method linear regression. BMD models were adjusted for age, fat-free mass, and BMI. The mean bilateral baPWV model was adjusted for age, right-brachial MAP, and BMI. B represents the change in the outcome per 10-point increase in tBPAQ. FDR q-values were calculated using the Benjamini–Hochberg procedure across the six primary raw *p*-values. BPAQ, Bone-Specific Physical Activity Questionnaire; BMD, bone mineral density; baPWV, brachial-ankle pulse wave velocity; CI, confidence interval.

## Data Availability

The datasets generated and/or analyzed in the current study are not publicly available to protect participants’ privacy but are available from the corresponding author upon reasonable request.

## Supplementary Materials

The following supporting information can be downloaded at: https://www.mdpi.com/article/10.3390/healthcare14193287/s1, Table S1. Distributional characteristics of BPAQ scores. Table S2. Covariate-adjusted bone mineral content by total BPAQ tertile. Table S3. Pearson, Spearman, and age-adjusted partial correlations between BPAQ and BMD/baPWV. Table S4. Secondary multivariable associations of current, past, and total BPAQ with primary outcomes. Table S5. Sensitivity analyses for the primary total-BPAQ models. Table S6. Side-specific baPWV sensitivity analyses. Table S7. Regression diagnostics and case-deletion sensitivity analyses for primary tBPAQ models. Table S8. Sensitivity of nominally significant quadratic terms to exclusion of the three highest tBPAQ observations. Figure S1A. Adjusted quadratic association between tBPAQ and femoral-neck BMD. Figure S1B. Adjusted quadratic association between tBPAQ and mean bilateral baPWV.
