# Supplementary material for: Association of Bone-Specific Physical Activity with Bone Mineral Density and Arterial Stiffness in Premenopausal Women: A Cross-Sectional Study
Source: Healthcare (Basel). 2026 Oct 2;14(19):3287. doi: 10.3390/healthcare14193287 (PMC13665021; doi:10.3390/healthcare14193287)
Supplement: Supplementary file 1 [file healthcare-14-03287-s001.zip › healthcare-4555564-supplementary.pdf]

Supplementary Material

Association of High-Impact Physical Activity with Bone Mineral Density and Arterial Stiffness in Premenopausal Women

Manuscript ID: healthcare-4555564

Table S1. Distributional characteristics of BPAQ scores

Variables	Mean	SD	Median	Q1	Q3	Skewness	KS D	p
cBPAQ	3.312	8.430	0.560	0.000	2.536	5.580	0.347	<.001
pBPAQ	19.526	23.279	10.237	2.470	31.087	1.929	0.202	<.001
tBPAQ	11.413	13.197	6.171	1.723	18.170	1.874	0.194	<.001

Note. Kolmogorov-Smirnov p-values use the Lilliefors correction. BPAQ, Bone-Specific Physical Activity Questionnaire.

Table S2. Covariate-adjusted bone mineral content by total BPAQ tertile

Variables	Low adjusted mean (95% CI)	Middle adjusted mean (95% CI)	High adjusted mean (95% CI)	F	p	FDR q	ηp^2
Whole-body BMC (kg)	2.08 (2.04–2.11)	2.07 (2.04–2.11)	2.16 (2.12–2.20)	6.676	.001	.002	.048
Femoral neck BMC (g)	3.74 (3.64–3.85)	3.71 (3.60–3.81)	3.98 (3.87–4.09)	7.102	<.001	.002	.051
Total femur BMC (g)	28.37 (27.65–29.09)	28.16 (27.44–28.88)	30.23 (29.49–30.96)	9.068	<.001	<.001	.064
Lumbar spine BMC (g)	59.12 (57.36–60.89)	59.99 (58.23–61.75)	62.65 (60.85–64.45)	3.953	.020	.020	.029
Forearm BMC (g)	11.87 (11.66–12.08)	11.65 (11.43–11.86)	12.15 (11.93–12.36)	5.160	.006	.008	.038

Note. General linear models used Type III sums of squares and adjusted for age, fat-free mass, and BMI. Pairwise estimated marginal mean comparisons were Bonferroni-adjusted; FDR q-values were calculated across the five BMC variables.

Table S3. Pearson, Spearman, and age-adjusted partial correlations between BPAQ and BMD/baPWV

BPAQ	Outcome	Pearson r	p	Spearman ρ	p	Age-adjusted partial r	p
cBPAQ	WBMD	0.055	.365	0.095	.121	0.059	.331
cBPAQ	FNBMD	0.148	.015	0.145	.017	0.118	.053
cBPAQ	FTBMD	0.106	.082	0.138	.023	0.081	.187
cBPAQ	LBMD	0.059	.331	0.086	.157	0.065	.286
cBPAQ	FaBMD	-0.034	.582	-0.023	.708	-0.016	.796
cBPAQ	RbaPWV	-0.062	.313	-0.126	.039	0.008	.895
cBPAQ	LbaPWV	-0.095	.120	-0.134	.028	-0.030	.627
cBPAQ	Mean baPWV	-0.080	.190	-0.130	.032	-0.012	.850
pBPAQ	WBMD	0.349	<.001	0.296	<.001	0.366	<.001
pBPAQ	FNBMD	0.429	<.001	0.295	<.001	0.391	<.001
pBPAQ	FTBMD	0.462	<.001	0.324	<.001	0.435	<.001
pBPAQ	LBMD	0.309	<.001	0.276	<.001	0.328	<.001
pBPAQ	FaBMD	0.174	.004	0.105	.085	0.214	<.001
pBPAQ	RbaPWV	-0.133	.029	-0.139	.023	-0.016	.793
pBPAQ	LbaPWV	-0.146	.016	-0.155	.011	-0.030	.619
pBPAQ	Mean baPWV	-0.142	.020	-0.151	.013	-0.024	.696
tBPAQ	WBMD	0.326	<.001	0.276	<.001	0.345	<.001
tBPAQ	FNBMD	0.426	<.001	0.281	<.001	0.386	<.001
tBPAQ	FTBMD	0.441	<.001	0.313	<.001	0.412	<.001
tBPAQ	LBMD	0.291	<.001	0.275	<.001	0.312	<.001
tBPAQ	FaBMD	0.143	.019	0.077	.206	0.184	.002
tBPAQ	RbaPWV	-0.136	.025	-0.145	.017	-0.011	.858
tBPAQ	LbaPWV	-0.159	.009	-0.170	.005	-0.036	.554
tBPAQ	Mean baPWV	-0.150	.013	-0.161	.008	-0.024	.690

Note. Partial correlations control for age only and are presented as a sensitivity analysis; multivariable linear regression is the primary adjusted analysis.

Table S4. Secondary multivariable associations of current, past, and total BPAQ with primary outcomes

Exposure	Outcome	B per 10 points	95% CI	Standardized β	p	Adjusted R^2
cBPAQ	Whole-body BMD	0.0023	-0.0082 to 0.0127	.024	.672	.141
cBPAQ	Femoral neck BMD	0.0160	-0.0004 to 0.0323	.107	.056	.189
cBPAQ	Total femur BMD	0.0098	-0.0044 to 0.0241	.077	.175	.175
cBPAQ	Lumbar spine BMD	0.0051	-0.0105 to 0.0207	.038	.518	.122
cBPAQ	Forearm BMD	-0.0013	-0.0062 to 0.0037	-0.029	.617	.116
cBPAQ	Mean bilateral baPWV	1.3	-13.3 to 15.8	.007	.865	.583
pBPAQ	Whole-body BMD	0.0110	0.0073 to 0.0146	.328	<.001	.241
pBPAQ	Femoral neck BMD	0.0188	0.0131 to 0.0244	.349	<.001	.291
pBPAQ	Total femur BMD	0.0186	0.0138 to 0.0234	.399	<.001	.318
pBPAQ	Lumbar spine BMD	0.0143	0.0088 to 0.0198	.289	<.001	.199
pBPAQ	Forearm BMD	0.0027	0.0009 to 0.0045	.174	.003	.143
pBPAQ	Mean bilateral baPWV	3.1	-2.4 to 8.5	.045	.269	.585
tBPAQ	Whole-body BMD	0.0178	0.0113 to 0.0244	.301	<.001	.224
tBPAQ	Femoral neck BMD	0.0331	0.0230 to 0.0431	.348	<.001	.290
tBPAQ	Total femur BMD	0.0314	0.0228 to 0.0400	.382	<.001	.304
tBPAQ	Lumbar spine BMD	0.0236	0.0137 to 0.0335	.271	<.001	.189
tBPAQ	Forearm BMD	0.0040	0.0008 to 0.0072	.145	.015	.134
tBPAQ	Mean bilateral baPWV	5.1	-4.6 to 14.7	.043	.301	.585

Note. Enter-method regression. BMD models were adjusted for age, fat-free mass, and BMI; mean bilateral baPWV models were adjusted for age, right-brachial MAP, and BMI. These analyses are exploratory secondary analyses and were not included in the primary six-outcome FDR family; nominal, unadjusted p-values are therefore reported and should be interpreted descriptively.

Table S5. Sensitivity analyses for the primary total-BPAQ models

Variables	log1p(tBPAQ) B (95% CI)	p	Centered linear B (95% CI)	p	Quadratic B (95% CI)	p	Adjusted R^2 (quadratic model)
Whole-body BMD	0.0173 (0.0091 to 0.0254)	<.001	0.0156 (0.0057 to 0.0256)	.002	0.00090 (-0.00216 to 0.00396)	.564	.222
Femoral neck BMD	0.0279 (0.0151 to 0.0406)	<.001	0.0202 (0.0051 to 0.0354)	.009	0.00526 (0.00060 to 0.00993)	.027	.300
Total femur BMD	0.0295 (0.0186 to 0.0404)	<.001	0.0234 (0.0103 to 0.0364)	<.001	0.00328 (-0.00072 to 0.00729)	.108	.308
Lumbar spine BMD	0.0261 (0.0139 to 0.0382)	<.001	0.0233 (0.0083 to 0.0383)	.002	0.00014 (-0.00448 to 0.00475)	.953	.186
Forearm BMD	0.0028 (-0.0011 to 0.0068)	.162	0.00265 (-0.00223 to 0.00752)	.286	0.00055 (-0.00095 to 0.00205)	.474	.133
Mean bilateral baPWV	-0.1767 (-11.8383 to 11.4848)	.976	-7.79 (-22.03 to 6.46)	.283	5.35 (0.94 to 9.75)	.017	.592

Note. The same covariates as the primary models were used. log1p(tBPAQ) is defined as ln(1+tBPAQ). Quadratic models used tBPAQ scaled per 10 points and mean-centered before construction of the squared term. Linear and quadratic coefficients shown in this table are from the same quadratic model. Adjusted R^2 therefore refers to the quadratic model. These nonlinear analyses are exploratory and were not included in the primary multiplicity-controlled analysis.

Table S6. Side-specific baPWV sensitivity analyses

Variables	Low adjusted mean (95% CI)	Middle adjusted mean (95% CI)	High adjusted mean (95% CI)	ANCOVA F	p	ηp^2	tBPAQ B per 10 points (95% CI)	Regression p
Right baPWV	1134.9 (1113.3–1156.5)	1131.3 (1109.9–1152.7)	1124.1 (1102.4–1145.7)	0.247	.781	.002	6.1 (-3.6 to 15.9)	.217
Left baPWV	1140.4 (1118.0–1162.7)	1128.1 (1105.9–1150.3)	1128.8 (1106.3–1151.3)	0.364	.695	.003	4.0 (-6.2 to 14.2)	.441

Note. ANCOVA and continuous regression models were adjusted for age, right-brachial MAP, and BMI. The primary vascular outcome in the manuscript is mean bilateral baPWV.

Table S7. Regression diagnostics and case-deletion sensitivity analyses for primary tBPAQ models

Variables	Max VIF	Max |studentized residual|	n > |3|	Max Cook's D	Primary B/10	Primary p	B/10 after excluding |residual|>3	Sensitivity p
Whole-body BMD	1.861	3.301	2	0.140	0.0178	<.001	0.0176	<.001
Femoral neck BMD	1.861	6.530	2	0.075	0.0331	<.001	0.0321	<.001
Total femur BMD	1.861	3.112	2	0.126	0.0314	<.001	0.0317	<.001
Lumbar spine BMD	1.861	3.285	1	0.114	0.0236	<.001	0.0241	<.001
Forearm BMD	1.861	3.440	2	0.075	0.0040	.015	0.0036	.024
Mean bilateral baPWV	1.351	4.098	2	0.055	5.1	.301	6.6	.158

Note. VIF, variance inflation factor. Primary coefficients are per 10-point increase in tBPAQ. One or two observations per model had |studentized residual|>3, but no Cook's distance approached 1. Case-deletion sensitivity analyses excluded only observations meeting the |studentized residual|>3 criterion; primary inference was unchanged.

Table S8. Sensitivity of nominally significant quadratic terms to exclusion of the three highest tBPAQ observations

Variables	Full-sample quadratic B	95% CI	p	Quadratic B after excluding top 3	95% CI	p	Top three tBPAQ values
Femoral neck BMD	0.00526	0.00060 to 0.00993	.027	0.00926	0.00298 to 0.01554	.004	69.632, 66.225, 65.112
Mean bilateral baPWV	5.35	0.94 to 9.75	.017	6.82	0.82 to 12.82	.026	69.632, 66.225, 65.112

Note. The same covariates as the corresponding primary models were retained. The persistence of the quadratic terms after exclusion of the three highest observed tBPAQ values suggests that the nominal findings were not attributable solely to those observations. These analyses remain exploratory and hypothesis-generating.

Figure S1A. Adjusted quadratic association between tBPAQ and femoral-neck BMD.

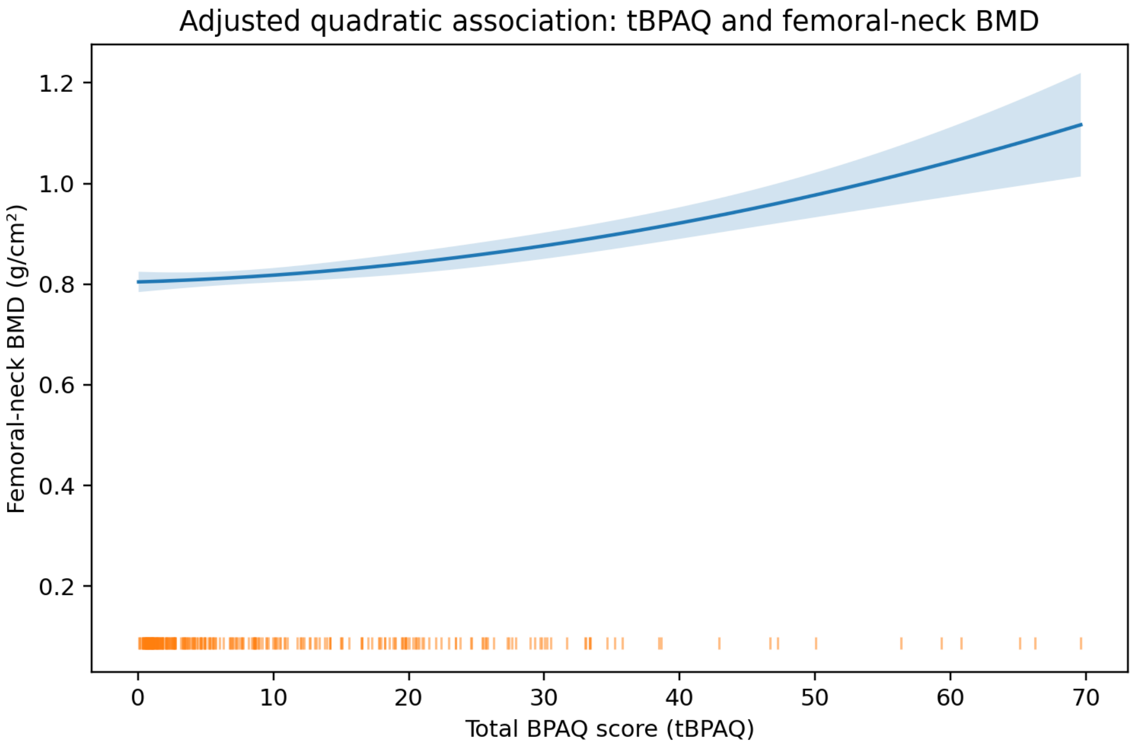

Note. Curves are adjusted for the covariates used in the corresponding primary model. Shaded areas represent 95% confidence intervals; rug marks show the observed tBPAQ distribution. Curves are exploratory and should not be interpreted as establishing nonlinear dose-response relationships.

Figure S1B. Adjusted quadratic association between tBPAQ and mean bilateral baPWV.

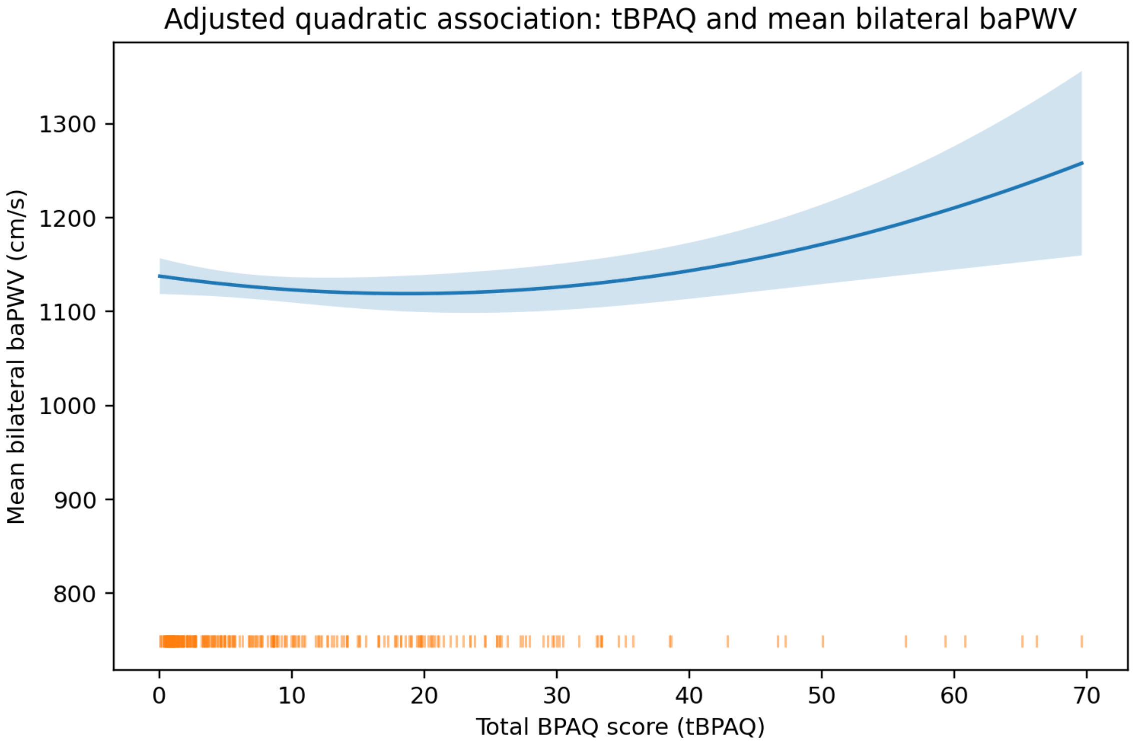

Note. Curves are adjusted for the covariates used in the corresponding primary model. Shaded areas represent 95% confidence intervals; rug marks show the observed tBPAQ distribution. Curves are exploratory and should not be interpreted as establishing nonlinear dose-response relationships.
